# Nut-cracking success and efficiency in two wild capuchin monkey populations

**DOI:** 10.1098/rsos.240161

**Published:** 2024-06-12

**Authors:** Tiago Falótico, Amanda C. Macedo, Matheus A. de Jesus, Tatiana Espinola, Tatiane Valença

**Affiliations:** ^1^ School of Arts, Sciences and Humanities, University of São Paulo, São Paulo, Brazil; ^2^ Capuchin Culture Project, Neotropical Primates Research Group, São Paulo, Brazil; ^3^ Technological Primates Research Group, Max Planck Institute for Evolutionary Anthropology, Leipzig, Germany; ^4^ Federal University of São Paulo, Diadema, Brazil; ^5^ Institute of Psychology, University of São Paulo, São Paulo, Brazil; ^6^ Department for the Ecology of Animal Societies, Max Planck Institute of Animal Behavior, Konstanz, Germany

**Keywords:** tool use, sex differences, tradition, population differences, hammerstones, camera trap

## Abstract

Capuchins can employ several strategies to deal with environmental challenges, such as using stone tools to access encapsulated resources. Nut-cracking is customary in several capuchin populations and can be affected by ecological and cultural factors; however, data on success and efficiency are only known for two wild populations. In this work, using camera traps, we assessed palm nut-cracking success and efficiency in two newly studied wild bearded capuchin populations (*Sapajus libidinosus*) and compared them with other sites. We tested the hypothesis that the overall success and efficiency of nut-cracking would be similar between sites when processing similar resources, finding partial support for it. Although using hammerstones of different sizes, capuchins had a similar success frequency. However, efficiency (number of strikes to crack a nut) was different, with one population being more efficient. We also tested whether success and efficiency varied between sexes in adults. We predict adult males would be more successful and efficient when cracking hard nuts. We found no differences between the sexes in one site but found sex differences in the other, although also for the low-resistant nut, which was unexpected. Our data add to the knowledge of capuchin nut-cracking behaviour flexibility, variance and potential cultural traits.

## 1. Introduction

Nut-cracking is one of the most ancient forms of tool use in primates. Evidence of stone tools for nut-cracking in the middle Pleistocene has been recorded in hominins’ archaeological sites [1]. Using tools to crack hard-shelled fruits allows the acquisition of otherwise inaccessible food resources or an increase in efficiency in opening the hard fruits.

Chimpanzees (*Pan troglodytes*), one of the most closely related extant species to humans, are observed cracking nuts in several populations [2–4]. While it is not a behaviour spread among all chimpanzees, it is a cultural variation in West African populations that has occurred for at least 4300 yr [5,6].

Long-tailed macaques (*Macaca fascicularis*) are the other catarrhine species that also nut-crack using stone tools to process sea almonds and oil palm nuts [7–9]. As in the chimpanzees, this behaviour is not observed in all populations but is primarily observed in coastal-dwelling populations [7,10], and some of them may have acquired this behaviour recently [11].

Capuchin monkeys are the only platyrrhine known to use tools for nut-cracking, mainly using stone tools. Although a few island-inhabitant white-faced capuchin (*Cebus imitator*) groups use stone tools to process nuts, crabs and snails [12,13], most observations of this behaviour are from wild populations of robust capuchin species (*Sapajus* spp.), mainly bearded capuchins, *S. libidinosus* [14–20].

Bearded capuchins living in savannah environments (Brazilian Cerrado and Caatinga biomes) have been observed for decades using stones to crack open different encased hard-to-access food resources, mainly palm nuts, but capuchins can also use tools to crack open fruits, cashew nuts, seeds, crabs and even to fragment other stones [20–23]. At least two populations of *S. libidinosus*, living at Serra da Capivara National Park (SCNP) and Ubajara National Park (UNP), have also been observed to customarily use stones for digging and using sticks as probes [24–26].

Nut-cracking behaviour in *S. libidinosus* is known to have occurred for at least 3000 yr in one of those populations (SCNP, [27]) but is probably much older [28] and can be considered a cultural behaviour in those populations [15,29].

The ontogeny of nut-cracking with stone tools in capuchins has been studied in captivity and natural conditions [30–33]. Capuchin monkey proficiency in using stone hammers and anvils varies with age and body mass, with proficiency being achieved between the ages of 2 and 5 yr, depending on the resource exploited [33,34]. In captivity, skills with object manipulation appear at around six months of age, at which time, the immatures have a repertoire of manipulating various objects on different surfaces, with the full repertoire of manipulation reached at 12 months [35]. In semi-free studies, capuchins from the age of 1 yr attempt to break nuts, even if they lack coordination and proper force to do so proficiently, but they can successfully crack nuts after 2 yr of age [34].

In the few studies that measure the success of nut-cracking by wild robust capuchins (percentage of nut-cracking events in which the nut was cracked), there are no significant differences in success in nut-cracking between sexes when dealing with low-resistance targets [19,21]. However, when trying to crack high-resistance palm nuts, which require the use of heavier hammerstones and more force to do so, larger individuals are more successful [19]. The capuchins in the well-studied population of Fazenda Boa Vista (FBV) had a success of approximately 90% for cracking open low-resistance resources (softer palm nuts) [19]. However, when dealing with high-resistance nuts at FBV, the males have a success of 90%, while the females have only 62% [19]. At SCNP, where the monkeys hammer only low-resistance targets with much smaller hammerstones, the success was 78%–98%, depending on the target, and there was no difference between the sexes [21]. Adult capuchin males are, on average, bigger than females [30], so we expect adult males to be more successful when nut-cracking high-resistance nuts but not when dealing with low-resistance nuts.

In this work, we aim to describe nut-cracking success and efficiency in two populations of wild bearded capuchins (*S. libidinosus*) that use stone tools for palm nut-cracking and compare them with the other sites from the literature. We will test the hypothesis that (i) the overall success (percentage of nuts cracked in nut-cracking events) and efficiency (number of hammerstone strikes for a successful nut-cracking) of processing palm nuts with stone tools will be similar between sites when monkeys are processing similar resources. We will also test whether (ii) the success and efficiency vary between sexes in adult individuals. We predict that adult males will be more successful (more nuts cracked per attempt) and efficient (fewer strikes to crack a nut) than females when cracking hard palm nuts but not when processing low-resistance nuts.

## 2. Material and methods

### 2.1. Study sites

**Figure 1.**
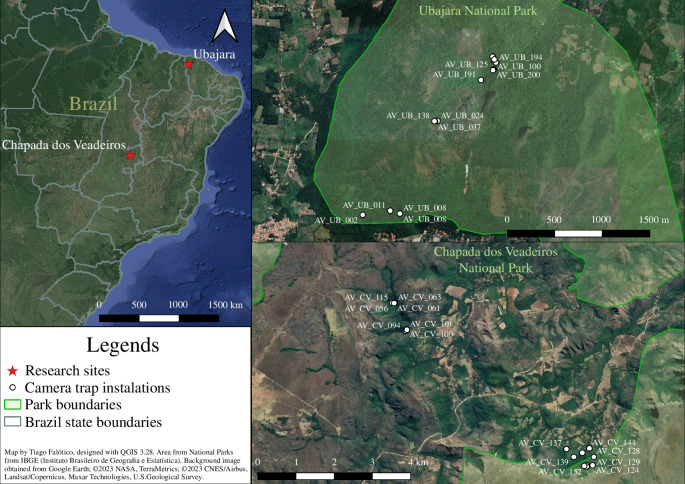
Maps of the research sites and the locations of the camera-trap installations.

Chapada dos Veadeiros National Park (CVNP) is located northeast of Goiás State (figure 1) in the Center-West region of Brazil. This site is located in the Cerrado biome and has a tropical savannah climate. The high altitude and high water availability enable a mosaic of vegetation, and the geomorphology includes granite gneiss, quartzites, conglomerates, calcium-pelitic rocks, sandstones, basalts, siltstone, limestones and dolostones (for more information, refer to [16]). We sampled two areas 5.2 km apart (Mariri and Terra Booma) that are home to at least one group of *S. libidinosus* each. Because the resources and lithic materials were the same, we analyse the two areas together in this work.

Ubajara National Park (UNP) is in Ceará State, Brazil (figure 1). This place has two different types of environments: the highland (900–700 m) and the lowland (below 400 m). The highland area presents higher annual rainfall (1459 mm) and lower average temperature (25.5°C), and it is classified as Tropical Seasonal Evergreen forest vegetation, with a higher density of *Attalea speciosa* palm trees, and a lower density of raw stone materials and nut-cracking sites. The lowland area presents lower annual rainfall (939 mm) and higher average temperature (27.1°C), a Stepic Savannah vegetation, a higher density of *Acrocomia aculeata* palm trees and a higher density of raw stone materials and nut-cracking sites. Between these areas (700–400 m), there is a transitional area with Tropical Seasonal Deciduous Forest vegetation; for more information, refer to [20].

### 2.2. Data collection

We installed camera traps at 36 nut-cracking sites (electronic supplementary material S1), 19 at CVNP (from 9 July to 2 December 2019), and 17 at UNP (from 7 October to 2 November 2020), sampling highland and lowland areas. The camera traps pointed to active nut-cracking sites identified in the previous mapping of processing sites [16,20]. The cameras were installed near the ground (up to 50 cm), parallel to the surface of the anvil and 1–3 m from the anvil. The cameras aimed to record the monkeys arriving and using the stones to process palm nuts (electronic supplementary material, S2). The anvils and hammers were previously marked with identification codes and measured (dimensions and weight) [16,20].

In October 2022, we collected a sample of 42 *Attalea* palm nuts from CVNP in four areas near nut-cracking sites where we observed capuchin nut-cracking, to test the nuts’ fracture resistance. The same sampling was done on Ubajara palm nuts in a previous study [20]. Whole nuts were collected from the ground. We tried to collect nuts without signs of parasites or that were not rotten. We peeled the mesocarp, if present, to store the dry sample for the trials. We aimed to compare the nut resistance across sites. The method used for measuring peak force to failure was the same as in previous studies, using a TONI COMP III universal tester at the Associação Brasileira de Cimento Portland, São Paulo, to test the nuts’ fracture resistance [20,36].

### 2.3. Coding

We defined a nut-cracking event to occur when a monkey approached an anvil and positioned a nut or fruit on top of the anvil. For this analysis, we included only the interactions of monkeys with single whole nuts; events that had interactions with more than one nut, half nuts and fragments were excluded (*Attalea* nuts have 4–7 alveoli with the kernels inside, and often those alveoli are not cracked at the same time, causing the monkeys to continue or begin to process half-broken nuts). We counted the number of times an individual struck the nut with the hammerstone (efficiency) and whether the nut was cracked (success). The event was considered over when the nut was opened (successful nut-cracking), even when we did not observe the nut being consumed (partial success), or if the individual left the anvil for more than 10 s without cracking the nut open (unsuccessful nut-cracking). We also noted the sex and age class and, when possible, individual ID. For this analysis, we considered the classes to be adults (greater than 5 yr) and immatures (less than 5 yr) based on morphological characteristics. We considered adults older than 5 yr to be sure they were correctly identified by morphological characteristics from the camera-trap footage and to be sure they were proficient nut-crackers. We also noted the hammers used in each event, as they had been marked and weighted during the previous mapping. During the coders’ training (A.C.M., M.A.d.J., T.E. and T.V.), we performed inter-observer reliability tests (Cohens’ kappa) with the software Boris 7.5 [37] until the coding of the analysed behaviours reached the threshold of 90% similarity across the coders.

### 2.4. Statistical analysis

For the statistical analysis, we included only events of adults processing a single whole target of *Attalea* or *Acrocomia* nuts (we excluded processing beginning with partly opened nuts and events from other resources that were not common). We only analysed events in which the monkeys used hammerstones with known weights.

We used generalized linear models (GLMs) and generalized linear mixed models (GLMMs) to compare the dependent variables, which were success (nut was cracked open), efficiency (number of strikes to open) and hammer weight (measured in grams). The independent variables were population, sex and target (food resource). For the analyses with sufficient sample sizes, we used the GLMM test to control for random variables (tool weight or sex, depending on the analysis). We used a Kruskal–Wallis (K–W) test to compare hammerstone weight by site and sex.

All the analyses were performed with *lme4* and *stats* packages in R [38]. The data and code used in the study are available as electronic supplementary material, S3 and S4.

## 3. Results

We registered 198.5 min of monkeys interacting with the nut-cracking sites at CVNP and 2880 min at UNP, totalling 1679 events of nut-cracking (details in table 1).

**Table 1.** Data collected with camera traps at the two sites and events analysed.

site	anvils sampled	camera-trap days	total events of nut-cracking	events with whole single nuts by adults
CVNP	19	2487	134	60
UNP	17	265	1545	967

**Figure 2.**
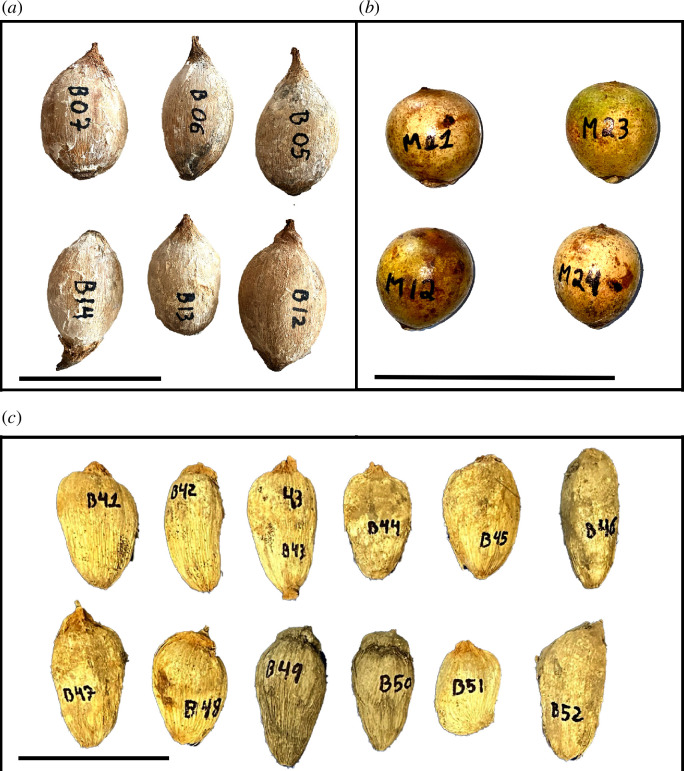
Palm nuts that capuchin monkeys (*S. libidinosus*) process with stones tools in the two areas. At UNP, (*a*) babaçu palm nut (*Attalea speciosa*) and (*b*) fresh macaúba palm nut (*Acrocomia aculeata*), and at CVNP, (*c*) palm nut from genus *Attalea*. The black line scales are 10 cm.

The nuts the monkeys were mostly observed to crack and that are being analysed here are an *Attalea* sp. palm nut at CVNP, and two palm nuts at UNP, babaçu (*Attalea speciosa*) and macaúba (*Acrocomia aculeata*). The monkeys were observed to crack other resources, such as jatobá (*Hymenaea courbaril*) and other fruits [16] but they did that at a very low frequency. We could not identify the CVNP palm nuts to the species level, but they are very similar in size and shape to the babaçu nut from UNP and could be the same or a sister species (e.g. *Attalea compta* or *A. brasiliensis*). From here on, we are going to refer to the palm nut from CVNP as *Attalea* and the palm nuts from UNP by their common names, babaçu and macaúba (figure 2).

Both larger nuts (*Attalea* and babaçu) are very similar in their resistance to fracture and about four times more resistant to cracking than macaúba nuts (table 2). We consider the *Attalea* and babaçu species as ‘high-resistance’ and the macaúba as ‘low-resistance’ for the analysis and discussion.

**Table 2.** Resistance, hammerstone weight average for the nut-cracking events, frequency of success of nut-cracking events by adult capuchin monkeys and efficiency (strikes to open resource) per food resource explored.

resource	site	resistance (peak-force-at-failure, kN) avg. ± s.d.	hammer weight (g) avg. ± s.d., range (*N*)	% success (*N*)	efficiency (strikes to open) avg. ± s.d., range (*N*)
palm nut (*Attalea* sp.)	CVNP	19.7 ± 7.47	2020.2 ± 971.3, 1228–4650 (60)	35% (60)	5.1 ± 7.4, 1–35 (21)
babaçu (*Attalea speciosa*)^a^	UNP	18.8 ± 3.90	1021.3 ± 15.2, 949–1024 (96)	37% (96)	15.8 ± 12.7, 3–58 (36)
macaúba (*Acrocomia aculeata*)^a^	UNP	4.15 ± 0.64	1473.1 ± 393.8, 259–2400 (871)	81% (871)	5.8 ± 4.1, 1–31 (704)

^a^ Peak-at-failure values for Babaçu and Macaúba from [20].

The success and efficiency of nut-cracking by resource and sex are presented in table 3.

**Table 3.** Sex differences in the frequency of success of nut-cracking events of adult capuchin monkeys, efficiency (strikes to open resource) and hammerstone weight (g) per resource explored in the events.

resource	site	sex	hammer weight (g) avg. ± s.d., range (*N*)	% success (*N*)	efficiency (strikes to open) avg. ± s.d., range (*N*)
palm nut (*Attalea* sp.)	CVNP	female	1796.3 ± 664.7, 1468–4400 (18)	39% (18)	7.7 ± 12.2, 1–35 (7)
		male	2116.1 ± 1069.1, 1228–4650 (42)	33% (42)	3.9 ± 3.0, 1–10 (14)
babaçu (*Attalea speciosa*)	UNP	female	1024.5 ± 0, 1024 (17)	12% (17)^a^	30 ± 29.7, 9–51 (2)^a^
		male	1020.7 ± 16.7, 949–1024 (79)	43% (79)^a^	14.9 ± 11.5, 3–58 (34)^a^
macaúba (*Acrocomia aculeata*)	UNP	female	1523.7 ± 410.8, 827–2400 (334)^a^	74% (334)^a^	6.0 ± 4.3, 1–27 (249)^a^
		male	1441.7 ± 379.9, 259–2400 (537)^a^	85% (537)^a^	5.6 ± 4.0, 1–31 (455)^a^

^a^ Significant difference between sexes. See the text for the statistical results.

### 3.1. Site differences

In general, the stones used by the capuchins in CVNP were heavier than the ones used at UNP (K–W, *χ* ^2^ = 26.204, *p* < 0.001, Cohen’s *d* = 0.798). Monkeys had a similar rate of success at cracking high-resistance nuts in both places (GLMM, *t* = 0.315, *p* = 0.7524), but their efficiency was higher at CVNP (GLMM, *t* = 11.757, *p* < 0.001, odds ratio = 3.824, CI (97.5%) = 4.782), maybe reflecting the heavier stone tools used.

### 3.2. Resources differences

Comparing the resources within the UNP capuchin population, we observed that monkeys had a lower percentage of success at cracking open babaçu (high resistance) compared with macaúba (low resistance; GLMM, *t* = 8.963, *p* < 0.001, odds ratio = 8.341, CI (97.5%) = 13.264), as expected. We also found that the macaúba was processed with heavier hammerstones (K–W, *χ* ^2^ = 89.708, *p* < 0.001, Cohen’s *d* = −1.62).

### 3.3. Sex differences

For the high-resistance resource at CVNP, we observed no sex differences in adult monkeys’ hammerstone weights used (K–W, *χ* ^2^ = 0.992, *p* < 0.319), success (GLM, *t* = –0.413, *p* = 0.68) or efficiency (GLM, *t* = 1.141, *p* = 0.254), although our sample for females was small for this population. The females were less frequently observed to process those nuts (table 3).

At UNP, females used heavier hammerstones (K–W, *χ* ^2^ = 12.558, *p* < 0.001, Cohen’s *d* = 0.207). Females were also less frequently observed to process high-resistance nuts (babaçu), were less successful (GLMM, *t* = 2.206, *p* = 0.0274, odds ratio = 5.666, CI (97.5%) = 26.432) and were less efficient than males when processing those nuts (a statistical test was not possible for efficiency because we only observed two events of females successfully cracking babaçu nuts). For the low-resistance nut (macaúba), males and females also differed in their success (GLMM, *t* = 3.852, *p* < 0.001, odds ratio = 2.034, CI (97.5%) = 2.919) and efficiency (GLMM, *t* = −4.035, *p* < 0.001, odds ratio = 0.867, CI (97.5%) = 0.929), with males being around 15% more successful and 7% more efficient.

## 4. Discussion

### 4.1. Sites and resources comparisons

The nut-cracking success between CVNP and UNP was similar for the high-resistance nuts, even if the capuchins at CVNP used heavier hammerstones to crack those nuts. This shows that capuchins adjust their behaviour to crack hard nuts in a similar fashion across populations. However, comparing the success of processing high-resistance nuts of those capuchins with the FBV population (table 4), we observed that FBV capuchins present much higher success. That could be because of a better technique used by FBV capuchins, bias in the data collection (e.g. including fragmented nuts or different nuts in the analysis), or, more likely, because the nuts that FBV capuchins process are easier to crack. Comparing the high-resistance nuts’ average peak-force-at-failure values for FBV, 8.2 and 11.5 kN [36], with the values at UNP (18.8 kN) and CVNP (19.7 kN), it is clear that the palm nuts from the sites analysed in the present study are much harder to break. We suggest that although the technique could be one of the reasons for the differences, the easier-to-crack resource is probably a better explanation. A controlled comparison with the same resource and similar hammerstone could be made in the future to compare nut-cracking techniques’ success in each population.

**Table 4.** Success and efficiency of nut-cracking for high and low-resistance nuts in wild capuchin monkeys (*S. libidinosus*) populations.

**population**	**h**igh-resistance nuts (**>7 kN** peak-force**-**at**-**failure)		**l**ow-resistance nuts (**<7 kN** peak-force**-**at**-**failure)		**references**
	**s**uccess (%)	**efficiency (strikes)**	**success (%)**	**efficiency (strikes)**	
Chapada dos Veadeiros	33–39%	3.9–7.7	—	—	this work
Ubajara	12–43%	15–30	74–85%	5.6–6	this work
Fazenda Boa Vista	62–90%	12–15	87–93%	8–13	[19]
Serra da Capivara	—	—	74–98%	—	[21]

Conversely, the efficiency (number of strikes to open the nut) significantly differed between CVNP and UNP, with the monkeys at the former being much more efficient. That could be because the hammerstones used at CVNP are heavier and also made of a more solid quartzite material [16]. The efficiency of FBV capuchins for high-resistance nut-cracking appears to fall close to the range of UNP, maybe slightly more efficient, but still much less efficient than CVNP capuchins cracking harder palm nuts. Both populations (FBV and UNP) also have a similar average hammerstone weight, which could help lead to the similarity in efficiency. There may be a balance between success and efficiency that each population reaches to continue exploiting those hard-to-break nuts cost-effectively over time. Comparing the use of low-resistance resources across populations (table 4), we observe less difference in success, with all populations reaching more than 74%, even reaching 98%.

In this case, the efficiency of processing the low-resistance target at UNP (macaúba nuts) is higher than at FBV. This may suggest that the capuchins at UNP are more specialized in processing this kind of resource; however, macaúba is slightly easier to crack (4.1 kN) than the low-resistance resources at FBV (5.1–5.6 kN) [36], so that could be the main reason for the differences.

When comparing the nut-cracking of low- and high-resistance nuts within UNP, we found, as expected, that the former is processed with much more success and efficiency. However, we also found that the low-resistance nuts were processed with heavier hammerstones, which was unexpected [39]. This might be related to the fact we had only sampled two sites with babaçu processing. Those were also located in the park’s highlands, an area with less stone material available [20].

Overall, our first hypothesis was not supported for high-resistance nuts. Success and efficiency were different when monkeys of distinct populations processed similar high-resistance food resources, indicating that other factors such as the range of nut resistance, average hammer weight, raw stone materials available and technique may play a role in the success and efficiency of nut-cracking, as already proposed in other studies [16,33,40]. For low-resistance nuts, however, the hypothesis was partially supported; maybe because those targets are less influenced by hammer size and composition, success is similar even if only lighter and less dense stones are available, although the efficiency can vary widely.

### 4.2. Sex differences

Our second hypothesis, that success and efficiency would vary between sexes in adult individuals, with males being more successful and efficient when cracking open high-resistance nuts because of their larger size, was not supported at CVNP, where male and female capuchins presented no differences in success and efficiency. Although females were less frequently observed to crack the high-resistance nuts, female efficiency had a large variance, overlapping with males. The sample was small, which could create a bias (see below); however, the monkeys in this population use, on average, the largest hammerstones ever observed for capuchins, average 1672.2 g [16], maybe allowing for the females to compensate for smaller body size [41].

The same hypothesis was partly supported at UNP. Females were less successful and less efficient in processing high-resistance targets. However, contrary to our predictions, females were also less successful and less efficient when processing low-resistance nuts. That is contrary to what was found in other studies, such as in FBV [19] and SCNP [21]. One explanation could be that our sample of events for low-resistance nuts is much larger (871 events) than that of FBV (129 events), meaning that our data could better identify statistically small differences between sexes. Adult males are, on average, larger and heavier than females [41], so extra force could produce a small difference even for low-resistance targets.

In the case of SCNP, the sample (1139 events) is similar to UNP, but the capuchins in that population process much softer resources (cashew nuts, *Manihot* seeds and *Cordia* fruits), which could be a reason for the absence of sex difference observed there [21]. Nevertheless, a more detailed analysis in the future could provide a better explanation for these conflicting results.

## 5. Conclusion

Capuchins are flexible primates, using several strategies to deal with challenges in their environments. The use of stone tools to access encapsulated resources is one of them. The success of using such tools can be impacted by the ecological factors of each area, such as the resistance to cracking of the targets and the availability of rocks. However, variability may also be due to the behaviour each population has in its repertoire, maintained by social learning over generations, creating cultures such as which stone to choose and where to use the tools.

Our results diverged in some points from the literature (e.g. mixed results on sex differences, with some results contrary to previous findings), showing that data from diverse locations are crucial to understanding the manifold variation of capuchin monkeys’ behaviour.

This is the first work about tool use in a population of robust capuchin monkeys that employed camera traps to identify details in the behaviours, such as success and efficiency, showing that this technology is promising for behaviour studies. With camera traps, it is possible to collect long-term data on nut-cracking site visits (not only by the monkeys but also by scavengers and predators), follow changes in stone selection and even check interactions between the individual monkeys, which are essential for understanding socially biased learning. Moreover, camera traps are especially interesting for producing quick data and comparing different populations, even unhabituated ones like the ones in the present study.

Our study added data from two populations to the literature on the success and efficiency of nut-cracking behaviour in robust capuchin monkeys, doubling the populations that have these data available and strengthening future comparisons.

## Data Availability

The data supporting this article are available as supplementary material [42].
